# Health Care Access and Affordability Among US Adults Aged 18 to 64 Years With Self-reported Post–COVID-19 Condition

**DOI:** 10.1001/jamanetworkopen.2023.7455

**Published:** 2023-04-10

**Authors:** Michael Karpman, Stephen Zuckerman, Sarah Morriss

**Affiliations:** 1 Health Policy Center, Urban Institute, Washington, District of Columbia

## Abstract

**Importance:**

Many US adults report having post–COVID-19 condition (PCC), but little is known about their access to health care.

**Objective:**

To estimate the association of PCC with access and affordability challenges among US adults aged 18 to 64 years.

**Design, Setting, and Participants:**

This survey study used data from the Health Reform Monitoring Survey, a probability-based internet survey conducted June 17 to July 5, 2022. Participants included a nationally representative sample of 9484 US adults ages 18 to 64 years drawn from the Ipsos KnowledgePanel.

**Main Outcomes and Measures:**

Self-reported PCC was defined as experiencing symptoms more than 4 weeks after first having COVID-19 that were not explained by another condition or factor. Access and affordability outcomes included having a usual place of care; forgoing care in the past 12 months because of costs, difficulty finding clinicians, or difficulty using health insurance; having problems paying family medical bills in the past 12 months; and having past-due medical debt.

**Results:**

Of 19 162 panel members recruited for the survey, 9599 individuals completed the survey (completion rate, 50.1%) and 9484 respondents were included in the final analytic sample (4720 females [50.6%, weighted]; mean [SD] age, 41.0 [13.5] years). A total of 3382 respondents (36.4%; 95% CI, 34.7%-38.2%) reported ever being diagnosed with COVID-19, among whom, 833 respondents (22.5%; 95% CI, 20.9%-24.2%) reported currently having PCC. After adjustment for differences in demographic, health, and geographic characteristics, adults with PCC were more likely than 2549 adults with a COVID-19 diagnosis but no report of PCC and 6102 adults never diagnosed with COVID-19 to report unmet health care needs in the past 12 months because of the following challenges: costs (27.0%; 95% CI, 23.2%-30.7% vs 18.3%; 95% CI, 15.9%-20.7% and 17.5%; 95% CI, 15.4%-19.6%) and difficulties finding clinicians accepting new patients (16.4%; 95% CI, 14.3%-18.4% vs 10.1%; 95% CI, 8.8%-11.5% and 10.7%; 95% CI, 9.6%-11.8%), getting a timely appointment (22.0%; 95% CI, 19.3%-24.8% vs 14.4%; 95% CI, 13.2%-15.7% and 13.9%; 95% CI, 12.9%-14.8%), and getting health plan care authorization (16.6%; 95% CI, 14.6%-18.6% vs 10.8%; 95% CI, 9.6%-12.1% and 10.3%; 95% CI, 9.4%-11.2%) (*P* < .001 for all comparisons).

**Conclusions and Relevance:**

This study found that adults aged 18 to 64 years with PCC were more likely than other adults to have difficulty getting and paying for health care. These findings suggest that policies aimed at improving access and affordability may focus on accelerating development of treatments and clinical guidelines, training clinicians, and addressing insurance-related administrative and cost barriers.

## Key Points

**Question:**

Are adults with post–COVID-19 condition (PCC) more likely than other adults to experience health care access and affordability challenges?

**Findings:**

In this survey study of 9484 US adults aged 18 to 64 years, a statistically higher rate of respondents with self-reported PCC did not obtain needed health care in the past year because of cost compared with adults without PCC. Adults with PCC were also more likely to have unmet needs because of difficulties getting timely appointments or health plan authorization, among other challenges with health care institutions or health insurance.

**Meaning:**

These findings suggest that improved health care access for adults with PCC may require developing clinical protocols and addressing insurance-related barriers.

## Teaser

This survey study examines the association of post–COVID-19 condition with the experience of health care access and affordability challenges among adults ages 18 to 64 years.

## Introduction

The persistence of symptoms after acute COVID-19 illness, or post–COVID-19 condition (PCC), poses a serious challenge to the US health care system. Early in the pandemic, patient support groups first identified this phenomenon as long COVID, which is also referred to as postacute sequelae of SARS-CoV-2 infection.^1^ Fatigue, shortness of breath, cognitive dysfunction, musculoskeletal pain, postexertional malaise, and loss of smell and taste are among the most common PCC symptoms,^2,3,4,5,6^ and studies have documented an increased risk of disorders of cardiovascular, neurologic, pulmonary, kidney, and other systems after the acute phase of infection.^7,8,9,10,11,12,13^ Definitions of PCC vary but generally refer to new or ongoing signs and symptoms occurring at least 4 weeks or 3 months after initial COVID-19 infection that cannot be explained by an alternative diagnosis, can affect any system of the body, and may relapse or worsen over time.^14,15,16^

Researchers have produced a wide range of estimates of the incidence of PCC among people with COVID-19 diagnoses. Retrospective studies in the US estimate that this incidence ranges from 5% to more than 20%, and a prospective cohort study in the Netherlands found an incidence of approximately 13%.^8,9,11,17^ The US Census Bureau’s July 27 to August 8, 2022, Household Pulse Survey found that approximately 17% of adults reported experiencing symptoms for more than 3 months that they did not have prior to infection.^18^ Current evidence suggests that the risk of developing PCC increases with the severity of the initial infection and is higher for women, people with chronic health conditions, and those who have not received a COVID-19 vaccination.^9,11,12,18,19,20,21,22^

The experiences of patients with similar and overlapping conditions, such as myalgic encephalomyelitis/chronic fatigue syndrome, suggest that US patients with PCC may experience difficulties navigating a fragmented health care system.^23,24,25,26^ Potential barriers to care include difficulty finding available and accessible clinicians, lack of care coordination, clinician attitudes toward or lack of knowledge about the illness, denial of health insurance claims for tests and treatments, and high out-of-pocket costs.^23,24,27^ The consequences associated with unmet medical needs may include exacerbated risk of disability and reduced health-related quality of life.^28^ Access to timely and effective treatment may be especially important for maintaining employment.^2,29^

To our knowledge, this is the first study examining health care access and affordability among working-age US adults with PCC. We used data from a nationally representative survey of adults ages 18 to 64 years conducted in June and July 2022 to assess differences in access between adults with self-reported PCC vs adults ever diagnosed with COVID-19 who did not report having PCC and adults who had never been diagnosed with COVID-19. We compared demographic, health, and geographic characteristics of these groups. We then investigated the association of self-reported PCC with the following access and affordability measures: having a usual place of care; unmet needs for care in the past year because of costs, difficulties finding a clinician, or difficulties using health insurance; problems paying medical bills in the past year; and past-due medical debt.

## Methods

The Urban Institute Institutional Review Board approved this survey study. Informed consent was obtained by the Ipsos KnowledgePanel when members joined the panel, and the survey included additional informed consent language. This study followed the American Association for Public Opinion Research (AAPOR) reporting guideline.^30^

Data used for this study were from the Health Reform Monitoring Survey, a national internet-based survey of adults aged 18 to 64 years conducted by the Urban Institute since 2013 that has been used to provide timely information on health care coverage and access.^31,32,33^ We conducted the survey for this study from June 17 to July 5, 2022. Respondents were sampled from the Ipsos KnowledgePanel, the largest US probability-based online research panel, with approximately 55 000 members. Panel members are recruited using address-based sampling methods from a sampling frame covering 97% of US households. These households are provided with free internet access and web-enabled devices if needed to facilitate their participation in the panel. The mean panel recruitment rate at the time the survey was conducted was 8.6%.

A stratified random sample of adults ages 18 to 64 years was selected from the panel, including oversamples of adults who were Asian, Black, Hispanic, or additional races other than White; ages 18 to 29 years; or in households with lower incomes (lower vs higher annual incomes defined as <250% vs ≥250% of the federal poverty level). Of 19 162 panel members assigned to the survey, 9599 individuals completed interviews, for a study completion rate of 50.1%. An additional 105 individuals were excluded owing to high levels of item nonresponse, and 10 individuals were excluded from analysis because they did not answer questions on diagnosed COVID-19 or PCC, yielding a final sample of 9484 adults. Nonrespondents were more likely than respondents to be ages 18 to 49 years, female, Black, Hispanic, and have lower educational attainment (respondents were more likely than nonrespondents to have attained a bachelor's degree, and nonrespondents were more likely to report a high school degree or less but no college attendance or some college attendance but no bachelor’s degree) (eTable 1 in Supplement 1). Survey design weights were used to adjust for unequal selection probabilities from the panel. Additionally, a poststratification raking procedure was used to adjust weights for nonresponse to produce representative estimates for the national adult population aged 18 to 64 years based on demographic, socioeconomic, and geographic benchmarks from the Current Population Survey and American Community Survey. Participants received an email with a link to the online survey, which was administered in English and Spanish and took a median (IQR) time of 13 (9-20) minutes to complete.

### Measures

Respondents were asked if a doctor or other health care professional ever told them that they had COVID-19 or if they ever took a test showing that they had COVID-19. Those who did were asked a modified question used in the UK Office for National Statistics Coronavirus Infection Survey: “Would you describe yourself as having ‘long COVID,’ that is, you are still experiencing symptoms more than 4 weeks after you first had coronavirus or COVID-19, that are not explained by something else?”^34^ The question was followed by a prompt providing examples of long-term symptoms derived from federal surveys.^35^ We categorized adults into 3 groups: those with current PCC, those who ever had a COVID-19 diagnosis but did not report current PCC, and those never diagnosed with COVID-19.

We assessed differences among these groups in several health care access outcomes, including whether they had a usual place of care. We also asked respondents if there was a time in the past 12 months when they needed the following types of care but did not receive them because they could not afford it: prescription drugs, care from a general doctor, care from a specialist, and medical tests, treatment, or follow-up care. Respondents who were ever insured in the past year were asked if there was a time they did not receive health care in the past 12 months because of difficulties getting health plan authorization for care or prescription drugs, finding a health care institution accepting their coverage, and getting information about their health plan network, covered services, or cost of care. Additional questions focused on unmet needs for care because of difficulties finding a clinician accepting new patients, getting to a doctor’s office when it was open, getting an appointment as soon as needed, getting a phone or video visit (ie, telehealth), and finding transportation. Finally, we asked respondents if they or their families had problems paying or were unable to pay medical bills in the past 12 months and if they currently had unpaid medical bills that were past due.

We used demographic data for sex, age, race and ethnicity, educational attainment, and citizenship status that were self-reported by respondents in a household profile questionnaire after joining the panel. Race and ethnicity data were collected and reported in accordance with US Office of Management and Budget standards.^36^ Respondents were classified as Hispanic or as non-Hispanic and Asian, Black, White, or another race and ethnicity, which included those who identified as American Indian or Alaska Native, Native Hawaiian or other Pacific Islander, more than 1 race, or some other race (these were combined into a single category owing to small sample sizes). Race and ethnicity data were collected from 2 separate questions, consistent with US Office of Management and Budget data-collection standards. However, we combined race and ethnicity (by including Hispanic individuals of all races in a single Hispanic category) when reporting estimates, which is consistent with how the US Census Bureau reports estimates for Hispanic and non-Hispanic individuals of various racial groups.^37^ Race and ethnicity were assessed due to longstanding racial and ethnic disparities in health care access and emerging evidence of racial and ethnic differences in rates of PCC. We also used panel data on residence in a rural area based on county of residence. The survey for this study collected additional self-reported information on marital status, presence of dependent children aged younger than 19 years in the household, whether a doctor or other health professional ever diagnosed respondents with selected physical conditions (hypertension; high cholesterol; heart disease; stroke; cancer; diabetes; asthma; chronic obstructive pulmonary disease, emphysema, or chronic bronchitis; and arthritis, gout, lupus, or fibromyalgia), and health insurance coverage status in the past year. The full survey instrument is available online.^38^

### Statistical Analysis

Estimates were weighted to be nationally representative of the adult population aged 18 to 64 years. We estimated the proportion of adults with self-reported PCC and the reported association of PCC with the ability to perform daily activities. We then compared differences in adult demographic, health, and geographic characteristics by the presence of self-reported COVID-19 diagnoses and PCC. We used multivariable logistic regression to estimate the association of self-reported PCC with health care access outcomes, controlling for differences in those characteristics. We did not control for coverage type (ie, private vs public insurance) or family income because of potential changes in these measures associated with PCC. Evidence suggests that PCC was associated with many adults not working and likely reduced income and access to employer-based insurance.^2,39,40^ However, after we controlled for these measures in sensitivity analyses, there was no meaningful change in results (eTables 2 and 3 in Supplement 1).

Probabilities of experiencing each outcome for each group of adults were estimated with a logistic regression model using the method of recycled predictions and the Stata margins command. This method used coefficients from the model to estimate outcomes for adults in the sample at a fixed value of the key explanatory variable (ie, all individuals were treated as having current PCC) while holding other covariates at their observed values, then finding the mean for those estimates over the sample. This process was repeated for all adults at other values of the key explanatory variable (ie, having COVID-19 but not PCC and never having COVID-19), allowing us to compare estimated probabilities after adjusting for differences across groups in other characteristics. Associations between PCC and access do not represent causal effects of PCC given that we could not observe and control for differences across groups that may have predated COVID-19 infection, such as differences associated with health status, access and affordability, or health insurance coverage. Moreover, no causal conclusions can be drawn from this observational study. Standard errors of estimated probabilities and differences between comparison groups account for the variance of coefficient estimates in the regression model. We report adjusted *P* values, which are *q* values, using the Benjamini-Hochberg correction to reduce the likelihood of type 1 errors when conducting multiple hypothesis tests.^41^ Statistically significant differences were those with an adjusted *P* value <.05 for 2-sided independent sample *t* tests. Analyses were conducted in Stata statistical software version 16.1 (StataCorp) using Stata’s survey commands and the poststratification weights; standard errors were clustered by state of residence to account for correlations within clusters. We conducted sensitivity analyses to test the robustness of the models with additional socioeconomic and geographic covariates and using bootstrapped standard errors (eTables 2-4 in Supplement 1).

## Results

There were 9484 respondents (4720 females [50.6%, weighted]; 356 Asian [6.3%], 1139 Black [12.7%], 1775 Hispanic [19.1%], 5744 White [58.9%], and 470 individuals with another race or ethnicity [3.0%]; mean [SD] age, 41.0 [13.5] years). A total of 3382 respondents, or more than one-third of respondents (36.4%; 95% CI, 34.7%-38.2%), reported having ever been diagnosed with COVID-19 (Table 1). Of respondents with a COVID-19 diagnosis, 833 individuals, or more than 1 in 5 individuals (22.5%; 95% CI, 20.9%-24.2%), reported currently having PCC symptoms lasting more than 4 weeks and 610 individuals, or nearly 1 in 6 individuals (16.2%; 95% CI, 14.5%-17.9%), reported such symptoms lasting more than 3 months. Overall, 8.2% (95% CI, 7.6%-8.8%) of adults reported PCC symptoms lasting at least 4 weeks. Most adults with PCC for this duration reported that their symptoms reduced their ability to carry out daily activities “a lot” (16.1%; 95% CI, 13.7%-18.4%) or “a little” (54.1%; 95% CI, 49.5%-58.6%) (Figure).

**Table 1.** Proportion of Adults Reporting COVID-19 Diagnosis and Current PCC^a^

COVID-19 status	Total, No (%) [95% CI] (N = 9484)	Ever diagnosed with COVID-19, % (95% CI) (n = 3382)
Ever told by doctor or other health professional that they had COVID-19 or ever tested positive for COVID-19	3382 (36.4) [34.7-38.2]	NA
Currently had PCC symptoms lasting >4 wk after first having COVID-19	833 (8.2) [7.6-8.8]	22.5 (20.9-24.2)
Currently had PCC symptoms lasting >3 mo after first having COVID-19	610 (5.9) [5.3-6.5]	16.2 (14.5-17.9)

Abbreviations: NA, not applicable; PCC, post–COVID-19 condition.

^a^ Sample sizes and counts of respondents are unweighted. Percentage estimates are weighted.

**Figure.**
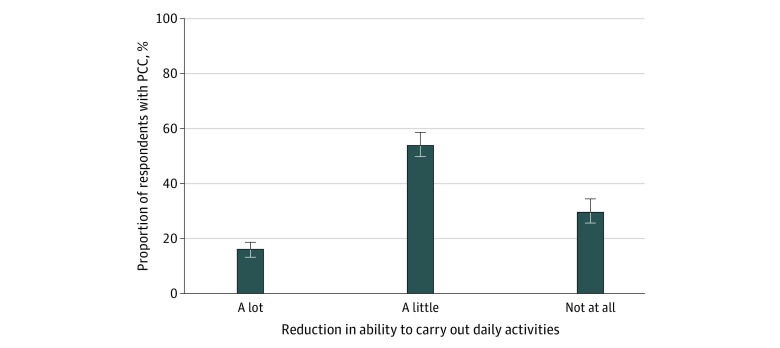
Reduced Ability to Carry Out Daily Activities Among Adults With Post–COVID-19 Condition (PCC) Respondents with self-reported PCC were asked whether their symptoms reduced their ability to carry out day-to-day activities compared with the time before they had COVID-19. Respondents could select 1 of the following options: yes, a lot; yes, a little; or not at all.

Adults reporting PCC were more likely to be female (65.4%; 95% CI, 61.5%-69.2%) than 2549 adults reporting a COVID-19 diagnosis but not PCC (48.3%; 95% CI, 45.6%-50.9%) and 6102 adults never diagnosed with COVID-19 (49.7%; 95% CI, 47.9%-51.5%) (Table 2). Adults with PCC were also more likely to be Hispanic (28.7%; 95% CI, 15.4%-42.1%) than adults in these other groups (18.7%; 95% CI, 10.2%-27.1% and 18.1%; 95% CI, 10.3%-25.9%, respectively) and have other chronic physical health conditions, results that are consistent with those of other surveys and studies.^9,11,18,19,22^ Nearly two-thirds of adults with PCC (62.9%; 95% CI, 59.2%-66.6%) reported that a clinician had ever diagnosed them with at least 1 of the surveyed physical health conditions. Adults with PCC were less likely to be insured in all months of the past year (81.9%; 95% CI, 77.3%-86.5%) than adults with COVID-19 diagnoses but not PCC (89.1%; 95% CI, 86.3%-92.0%) and had similar past-year coverage status as adults never diagnosed with COVID-19 (82.3%; 95% CI, 78.4%-86.3%). Residence in a rural area did not differ significantly across groups.

**Table 2.** Demographic and Health Characteristics

Characteristic	Adults, No. (%) [95% CI]^a^ (N = 9484)		
	With current PCC (n = 833)	Ever diagnosed with COVID-19 but without current PCC (n = 2549)	Never diagnosed with COVID-19 (n = 6102)
Sex			
Female	533 (65.4) [61.5-69.2]	1230 (48.3) [45.6-50.9]	2957 (49.7) [47.9-51.5]
Male	300 (34.6) [30.8-38.5]	1319 (51.7) [49.1-54.4]	3145 (50.3) [48.5-52.1]
Age, y			
18-34	198 (34.0) [30.3-37.7]	736 (39.2) [36.6-41.8]	1517 (37.2) [35.8-38.6]
35-49	305 (34.2) [30.3-38.1]	830 (31.3) [28.6-33.9]	1909 (29.5) [28.3-30.7]
50-64	330 (31.8) [28.9-34.6]	983 (29.5) [27.5-31.5]	2676 (33.3) [32.1-34.5]
Race and ethnicity			
Asian	9 (1.6) [0.2-3.0]	82 (5.7) [3.6-7.8]	265 (7.2) [2.9-11.4]
Black	69 (11.0) [7.0-15.0]	232 (9.5) [7.1-11.9]	838 (14.4) [11.1-17.7]
Hispanic	231 (28.7) [15.4-42.1]	498 (18.7) [10.2-27.1]	1046 (18.1) [10.3-25.9]
White	475 (55.6) [43.3-68.0]	1619 (63.4) [55.0-71.8]	3650 (57.3) [48.1-66.5]
Other^b^	49 (3.0) [1.9-4.1]	118 (2.7) [1.8-3.7]	303 (3.1) [2.3-3.9]
Educational attainment			
≤High school degree	269 (38.3) [33.8-42.8]	696 (32.6) [30.1-35.1]	2017 (38.4) [36.1-40.6]
Some college	288 (32.8) [28.9-36.7]	768 (29.2) [26.8-31.5]	1795 (26.3) [24.5-28.1]
≥Bachelor's degree	276 (28.9) [25.7-32.2]	1085 (38.3) [34.9-41.6]	2290 (35.3) [33.0-37.7]
Citizen	769 (91.9) [87.7-96.2]	2421 (94.9) [92.4-97.3]	5776 (93.4) [90.2-96.6]
Married	443 (52.5) [48.9-56.1]	1459 (55.2) [52.7-57.7]	3013 (49.4) [47.4-51.5]
Living with dependent children aged <19 y	333 (41.7) [38.0-45.4]	890 (36.5) [33.5-39.5]	1754 (31.8) [30.0-33.6]
Diagnosed physical health condition			
Hypertension	324 (33.8) [28.1-39.5]	658 (21.5) [19.5-23.6]	1748 (22.6) [20.7-24.4]
High cholesterol	267 (27.2) [23.8-30.5]	654 (22.3) [19.8-24.8]	1632 (22.3) [21.1-23.4]
Coronary heart disease, angina, heart attack, or other heart condition	84 (8.8) [6.7-10.9]	107 (3.0) [2.3-3.8]	327 (4.0) [3.3-4.6]
Stroke	19 (2.0) [1.2-2.8]	37 (1.2) [0.8-1.6]	97 (1.1) [0.8-1.4]
Cancer	67 (7.5) [5.7-9.3]	129 (4.3) [3.4-5.2]	312 (4.1) [3.5-4.8]
Diabetes	132 (14.4) [10.9-17.8]	252 (7.9) [6.6-9.3]	653 (8.1) [7.6-8.7]
Asthma	173 (20.5) [17.1-23.9]	311 (12.7) [11.0-14.5]	773 (11.7) [10.7-12.7]
COPD, emphysema, or chronic bronchitis	67 (6.8) [5.1-8.5]	58 (1.8) [1.3-2.3]	252 (2.8) [2.2-3.4]
Arthritis, gout, lupus, or fibromyalgia	223 (22.1) [18.8-25.5]	365 (11.0) [9.7-12.4]	1116 (13.6) [12.1-15.2]
Any of these physical health conditions	568 (62.9) [59.2-66.6]	1359 (49.1) [46.5-51.7]	3312 (46.7) [44.8-48.5]
Multiple physical health conditions	354 (36.5) [32.3-40.7]	661 (21.2) [19.5-22.9]	1900 (24.5) [22.8-26.2]
Health insurance coverage in past year^c^			
Insured all months	695 (81.9) [77.3-86.5]	2253 (89.1) [86.3-92.0]	5064 (82.3) [78.4-86.3]
Insured some but not all months	89 (12.3) [9.4-15.1]	159 (6.3) [4.8-7.8]	479 (8.1) [7.1-9.1]
Uninsured all months	48 (5.8) [2.6-9.1]	132 (4.6) [2.9-6.4]	530 (9.5) [6.4-12.7]
Residence in a rural area^d^	136 (13.9) [9.5-18.4]	357 (12.1) [8.2-15.9]	845 (12.3) [8.4-16.2]

Abbreviations: COPD, chronic obstructive pulmonary disease; PCC, post–COVID-19 condition.

^a^ Sample sizes and counts of respondents are unweighted. Percentage estimates are weighted.

^b^ Other race and ethnicity includes adults who did not identify as Hispanic and who identified as American Indian or Alaska Native, Native Hawaiian or other Pacific Islander, more than 1 race, or some other race.

^c^ Estimates exclude respondents with missing information on health insurance coverage in the past year.

^d^ Adults who lived in a rural area include those who did not live in a metropolitan statistical area.

Self-reported PCC did not have an association with having a usual place of care after adjustment for differences in demographic and geographic characteristics, insurance status, and health conditions (Table 3). However, adults with PCC were more likely than those with a COVID-19 diagnosis but not PCC and those never diagnosed with COVID-19 to report unmet health care needs in the past year because of costs (27.0%; 95% CI, 23.2%-30.7% vs 18.3%; 95% CI, 15.9%-20.7% and 17.5%; 95% CI, 15.4%-19.6%; *P* < .001 for both comparison) and because of difficulties finding a clinician accepting new patients (16.4%; 95% CI, 14.3%-18.4% vs 10.1%; 95% CI, 8.8%-11.5% and 10.7%; 95% CI, 9.6%-11.8%; *P* < .001 for both comparisons), getting an appointment as soon as needed (22.0%; 95% CI, 19.3%-24.8% vs 14.4%; 95% CI, 13.2%-15.7% and 13.9%; 95% CI, 12.9%-14.8%; *P* < .001 for both comparisons), getting to a doctor’s office or clinic when it was open (11.0%; 95% CI, 8.8%-13.2% vs 5.3%; 95% CI, 4.5%-6.2% and 6.7%; 95% CI, 6.2%-7.2%; *P* < .001 for both comparison), and getting a telehealth visit (7.6%; 95% CI, 5.4%-9.8% vs 3.1%; 95% CI, 2.5%-3.8% and 4.0%; 95% CI, 3.3%-4.7%; *P* < .001 for both comparisons). Adults with PCC were more likely than other adults with a COVID-19 diagnosis to have forgone care because of difficulty finding transportation (6.1%; 95% CI, 4.3%-8.0% vs 3.5%; 95% CI, 2.7%-4.3%; *P* = .01) but were not significantly more likely to report this experience than adults never diagnosed with COVID-19 (5.1%; 95% CI, 4.3%-5.9%; *P* = .38). Nearly 1 in 4 adults with PCC reported problems paying family medical bills in the past 12 months (23.5%; 95% CI, 19.4%-27.7%) and past-due medical debt (23.8%; 95% CI, 18.8%-28.9%) compared with fewer than 1 in 6 adults without PCC (Table 3).

**Table 3.** Health Care Access Challenges Experienced

Challenge	Adults with current PCC (n = 833)	Adults ever diagnosed with COVID-19 but without current PCC (n = 2549)		Adults never diagnosed with COVID-19 (n = 6102)	
	Adjusted, % (95% CI)^a^	Adjusted, % (95% CI)^a^	Adjusted P value^b^	Adjusted, % (95% CI)^a^	Adjusted P value^b^
Has a usual place to get health care	82.8 (79.5-86.1)	83.5 (81.2-85.8)	.73	80.3 (78.3-82.3)	.22
Did not get needed health care in past 12 mo because of costs	27.0 (23.2-30.7)	18.3 (15.9-20.7)	<.001	17.5 (15.4-19.6)	<.001
Did not get needed health care in past 12 mo because of difficulties finding clinician					
Finding a clinician accepting new patients	16.4 (14.3-18.4)	10.1 (8.8-11.5)	<.001	10.7 (9.6-11.8)	<.001
Getting an appointment as soon as needed	22.0 (19.3-24.8)	14.4 (13.2-15.7)	<.001	13.9 (12.9-14.8)	<.001
Getting to a doctor's office or clinic when it was open	11.0 (8.8-13.2)	5.3 (4.5-6.2)	<.001	6.7 (6.2-7.2)	<.001
Getting a telehealth visit	7.6 (5.4-9.8)	3.1 (2.5-3.8)	<.001	4.0 (3.3-4.7)	<.001
Finding transportation	6.1 (4.3-8.0)	3.5 (2.7-4.3)	.01	5.1 (4.3-5.9)	.38
Problems paying family medical bills in past 12 mo	23.5 (19.4-27.7)	15.9 (13.4-18.3)	<.001	14.7 (12.7-16.7)	<.001
Currently has medical bills that are past due	23.8 (18.8-28.9)	15.7 (12.4-19.0)	<.001	14.0 (11.6-16.5)	<.001

Abbreviation: PCC, post–COVID-19 condition.

^a^ Percentage estimates reflect estimated probabilities from a multivariable logistic regression controlling for differences in sex, age, race and ethnicity, educational attainment, citizenship status, marital status, presence of dependent children younger than age 19 years in the household, diagnosed physical health conditions, health insurance coverage status in the past 12 months, and residence in a rural area.

^b^ *P* values were estimated using independent sample *t* tests of the difference from adults with PCC and were adjusted using the Benjamini-Hochberg correction for multiple tests.

Among adults insured in the past year, those with PCC reported more difficulties using their health plans (Table 4). They were more likely than adults with a COVID-19 diagnosis but not PCC and adults never diagnosed with COVID-19 to report unmet care needs because of difficulties getting health plan authorization (16.6%; 95% CI, 14.6%-18.6% vs 10.8%; 95% CI, 9.6%-12.1% and 10.3%; 95% CI, 9.4%-11.2%), finding a doctor accepting their coverage (15.9%; 95% CI, 13.4%-18.3% vs 9.6%; 95% CI, 8.4%-10.9% and 10.0%; 95% CI, 9.0%-11.0%), and getting information from their plan about health care networks, covered services, or cost of care (13.6%; 95% CI, 11.1%-16.2% vs 7.6%; 95% CI, 6.5%-8.6% and 8.6%; 95% CI, 7.3%-9.9%) (*P* for all comparisons < .001]). We observed similar results in sensitivity tests controlling for coverage type, family income, census region, and population to primary care physician ratio (eTables 2-3 in Supplement 1).

**Table 4.** Unmet Needs for Medical Care Because of Difficulties Using Health Insurance

Unmet need^a^	Adults with current PCC (n = 785)	Adults ever diagnosed with COVID-19 but without PCC (n = 2416)		Adults never diagnosed with COVID-19 (n = 5562)	
	Adjusted, % (95% CI)^b^	Adjusted, % (95% CI)^b^	Adjusted P value^c^	Adjusted, % (95% CI)^b^	Adjusted P value^c^
Getting authorization for care or prescription drugs	16.6 (14.6-18.6)	10.8 (9.6-12.1)	<.001	10.3 (9.4-11.2)	<.001
Finding a doctor accepting coverage type	15.9 (13.4-18.3)	9.6 (8.4-10.9)	<.001	10.0 (9.0-11.0)	<.001
Getting information from plan on clinicians in network, covered services, or cost of care	13.6 (11.1-16.2)	7.6 (6.5-8.6)	<.001	8.6 (7.3-9.9)	<.001

Abbreviation: PCC, post–COVID-19 condition.

^a^ Unmet needs refer to health care needs that the individual did not receive in the past 12 months because of difficulties using health insurance.

^b^ Percentage estimates reflect estimated probabilities from a multivariable logistic regression controlling for differences in sex, age, race and ethnicity, educational attainment, citizenship status, marital status, presence of dependent children younger than age 19 years in the household, diagnosed physical health conditions, health insurance coverage status in the past 12 months, and residence in a rural area.

^c^ *P* values were estimated using independent samples *t* tests of the difference from adults with PCC and were adjusted using the Benjamini-Hochberg correction for multiple tests.

## Discussion

In this survey study, more than 1 in 5 adults with a COVID-19 diagnosis reported lasting symptoms more than 4 weeks after the initial acute infection, and most of these adults reported that PCC had at least some impact on their daily activities. After we controlled for demographic, geographic, and health differences, adults with PCC were more likely than other adults to report a range of health care access and affordability challenges.

Although most adults with PCC had a usual source of care, some may have had trouble finding clinicians who were knowledgeable about and responsive to their conditions.^42^ In addition, some patients may not have been aware of or had access to a multidisciplinary PCC clinic. Because PCC is an emerging chronic condition, many clinicians may be constrained by a lack of evidence on effective diagnostic tests and treatments, which may be associated with increased likelihood of referring patients to 1 or more specialists with limited availability. Despite substantial federal investments in research to identify treatments for PCC, these initiatives have been criticized for insufficient urgency and coordination.^43^ Accelerated progress in identifying treatments and disseminating clinical guidance may help more clinicians manage patient symptoms and better coordinate care.

Insurance-related barriers may also reflect limited evidence to inform standards of care. Insurers may deny reimbursement for tests and procedures that they do not consider medically necessary.^44^ Prior authorization and other use-management practices may also be associated with claim denials and other administrative burdens for patients.^45,46^ Insurance regulators and self-insured employers may potentially set standards for these practices to reduce delays in access to tests and treatments.

Even when services were covered by insurance, increased medical expenditures in the months after acute COVID-19 illness^47^ were likely to expose patients with PCC to higher out-of-pocket costs. Although federal law required health plans to cover COVID-19 testing and some treatments without cost sharing during the public health emergency,^48^ these requirements extended to treatment of PCC only for Medicaid plans, and the public health emergency will expire in May 2023.^49^ Limiting cost sharing for PCC and other chronic illnesses beyond the public health emergency could reduce medical debt and forgone care by spreading the risk of high costs more broadly across the population. In addition, further research is needed to gain a deeper understanding of patient experiences in seeking care for PCC symptoms, monitor whether access challenges persist over time, and assess social, economic, and health outcomes associated with unmet needs for care.

### Limitations

Our analysis has several limitations. Survey weights may mitigate but not eliminate nonresponse error and other sampling biases. Reported access challenges in the past 12 months were subject to recall bias.

COVID-19 diagnoses and PCC were self-reported. The survey may have misclassified some adults by restricting the group with PCC to respondents with a COVID-19 diagnosis or positive test. Some respondents who were not diagnosed could have had a COVID-19 infection, and a subset of them may have experienced PCC symptoms. Underdiagnosis of COVID-19 may be associated with a lack of available tests^50^ or limited health care access and coverage, which would reduce outcomes showing the estimated association between PCC and unmet care needs. This may be offset if other respondents misidentified unrelated symptoms as PCC. Representative surveys may provide evidence that is unavailable from administrative sources, such as insurance claims or electronic health records, which have also been found to have limitations because of clinical uncertainty, limited diagnostic testing and coding of PCC, and exclusion of people not receiving care.^50,51,52,53^

Associations between PCC and health care access do not imply causality, and no causal conclusions can be drawn from this observational study. We do not know if forgone care and medical bill problems were for PCC or other health conditions or if health care access barriers predated COVID-19 illness. Longitudinal data are needed to understand how these outcomes changed over time for individuals who developed PCC. Although we controlled for observed health characteristics, some chronic conditions in our model may have been brought on by PCC or associated with increased risk of developing PCC. COVID-19–related medical needs may also have been associated with changes in health insurance coverage status in the past year. There may be other unobserved differences between adults with and without PCC that explain differences in access challenges, but this would not alter the key finding that adults with PCC had increased risk of experiencing barriers to care.

## Conclusions

In this survey study of US adults aged 18 to 64 years that was conducted in June and July 2022, adults with self-reported PCC experienced greater challenges with health care access and affordability than other adults. These barriers may be associated with adverse outcomes for workforce participation and long-term health. Policy makers may be able to expand access to care by accelerating research on PCC treatments, disseminating clinical care guidelines, and regulating insurance practices.

## Notes

Supplement 1. **eTable 1.** Demographic and Geographic Characteristics of Health Reform Monitoring Survey Respondents and Nonrespondents **eTable 2.** Logistic Regression Models Estimating Association of Post–COVID-19 Condition With Unmet Needs for Health Care in Past 12 Months Because of Costs **eTable 3.** Estimated Probabilities of Health Care Access Outcomes **eTable 4.** Estimated Probabilities of Health Care Access Outcomes Using Bootstrapped Standard Errors
eReferences.

Supplement 2.
Data Sharing Statement

## References

[1] Callard F, Perego E. How and why patients made long COVID. Soc Sci Med. 2021;268:113426. doi:10.1016/j.socscimed.2020.113426 33199035 PMC7539940

[2] Davis HE, Assaf GS, McCorkell L, . Characterizing long COVID in an international cohort: 7 months of symptoms and their impact. EClinicalMedicine. 2021;38:101019. doi:10.1016/j.eclinm.2021.101019 34308300 PMC8280690

[3] Logue JK, Franko NM, McCulloch DJ, . Sequelae in adults at 6 months after COVID-19 infection. JAMA Netw Open. 2021;4(2):e210830. doi:10.1001/jamanetworkopen.2021.0830 33606031 PMC7896197

[4] Groff D, Sun A, Ssentongo AE, . Short-term and long-term rates of postacute sequelae of SARS-CoV-2 infection: a systematic review. JAMA Netw Open. 2021;4(10):e2128568. doi:10.1001/jamanetworkopen.2021.28568 34643720 PMC8515212

[5] Ayoubkhani D, Khunti K, Nafilyan V, . Post-covid syndrome in individuals admitted to hospital with COVID-19: retrospective cohort study. BMJ. 2021;372(693):n693. doi:10.1136/bmj.n693 33789877 PMC8010267

[6] Whittaker HR, Gulea C, Koteci A, . GP consultation rates for sequelae after acute COVID-19 in patients managed in the community or hospital in the UK: population based study. BMJ. 2021;375:e065834. doi:10.1136/bmj-2021-065834 34965929 PMC8715128

[7] Lopez-Leon S, Wegman-Ostrosky T, Perelman C, . More than 50 long-term effects of COVID-19: a systematic review and meta-analysis. Sci Rep. 2021;11(1):16144. doi:10.1038/s41598-021-95565-8 34373540 PMC8352980

[8] Bull-Otterson L, Baca S, Saydah S, . Post-COVID conditions among adult COVID-19 survivors aged 18-64 and ≥65 years—United States, March 2020-November 2021. MMWR Morb Mortal Wkly Rep. 2022;71:713-717. doi:10.15585/mmwr.mm7121e1 PMC9368731 35925799

[9] Daugherty SE, Guo Y, Heath K, . Risk of clinical sequelae after the acute phase of SARS-CoV-2 infection: retrospective cohort study. BMJ. 2021;373(1098):n1098. doi:10.1136/bmj.n1098 34011492 PMC8132065

[10] Al-Aly Z, Xie Y, Bowe B. High-dimensional characterization of post-acute sequelae of COVID-19. Nature. 2021;594(7862):259-264. doi:10.1038/s41586-021-03553-9 33887749

[11] Xie Y, Bowe B, Al-Aly Z. Burdens of post-acute sequelae of COVID-19 by severity of acute infection, demographics and health status. Nat Commun. 2021;12(1):6571. doi:10.1038/s41467-021-26513-3 34772922 PMC8589966

[12] Taquet M, Dercon Q, Luciano S, Geddes JR, Husain M, Harrison PJ. Incidence, co-occurrence, and evolution of long-COVID features: a 6-month retrospective cohort study of 273,618 survivors of COVID-19. PLoS Med. 2021;18(9):e1003773. doi:10.1371/journal.pmed.1003773 34582441 PMC8478214

[13] Taquet M, Sillett R, Zhu L, . Neurological and psychiatric risk trajectories after SARS-CoV-2 infection: an analysis of 2-year retrospective cohort studies including 1 284 437 patients. Lancet Psychiatry. 2022;9(10):815-827. doi:10.1016/S2215-0366(22)00260-7 35987197 PMC9385200

[14] Soriano JB, Murthy S, Marshall JC, Relan P, Diaz JV; WHO Clinical Case Definition Working Group on Post-COVID-19 Condition. A clinical case definition of post-COVID-19 condition by a Delphi consensus. Lancet Infect Dis. 2022;22(4):e102-e107. doi:10.1016/S1473-3099(21)00703-9 34951953 PMC8691845

[15] Venkatesan P. NICE guideline on long COVID. Lancet Respir Med. 2021;9(2):129. doi:10.1016/S2213-2600(21)00031-X 33453162 PMC7832375

[16] US Department of Health and Human Services, Office of the Assistant Secretary for Health. National research action plan on long COVID. Accessed August 17, 2022. https://www.covid.gov/assets/files/National-Research-Action-Plan-on-Long-COVID-08012022.pdf

[17] Ballering AV, van Zon SKR, Olde Hartman TC, Rosmalen JGM; Lifelines Corona Research Initiative. Persistence of somatic symptoms after COVID-19 in the Netherlands: an observational cohort study. Lancet. 2022;400(10350):452-461. doi:10.1016/S0140-6736(22)01214-4 35934007 PMC9352274

[18] Centers for Disease Control and Prevention. Long COVID: household pulse survey. Accessed August 20, 2022. https://www.cdc.gov/nchs/covid19/pulse/long-covid.htm

[19] Ioannou GN, Baraff A, Fox A, . Rates and factors associated with documentation of diagnostic codes for long COVID in the national Veterans Affairs health care system. JAMA Netw Open. 2022;5(7):e2224359. doi:10.1001/jamanetworkopen.2022.24359 35904783 PMC9338411

[20] Al-Aly Z, Bowe B, Xie Y. Long COVID after breakthrough SARS-CoV-2 infection. Nat Med. 2022;28(7):1461-1467. doi:10.1038/s41591-022-01840-0 35614233 PMC9307472

[21] Azzolini E, Levi R, Sarti R, . Association between BNT162b2 vaccination and long COVID after infections not requiring hospitalization in health care workers. JAMA. 2022;328(7):676-678. doi:10.1001/jama.2022.11691 35796131 PMC9250078

[22] Ayoubkhani D, Bermingham C, Pouwels KB, . Trajectory of long COVID symptoms after COVID-19 vaccination: community based cohort study. BMJ. 2022;377:e069676. doi:10.1136/bmj-2021-069676 35584816 PMC9115603

[23] Lin JMS, Brimmer DJ, Boneva RS, Jones JF, Reeves WC. Barriers to healthcare utilization in fatiguing illness: a population-based study in Georgia. BMC Health Serv Res. 2009;9:13. doi:10.1186/1472-6963-9-13 19154587 PMC2651135

[24] Thanawala S, Taylor RR. Service utilization, barriers to service access, and coping in adults with chronic fatigue syndrome. J Chronic Fatigue Syndr. 2007;14(1):5-21. doi:10.1300/J092v14n01_02

[25] Phillips S, Williams MA. Confronting our next national health disaster—long-haul COVID. N Engl J Med. 2021;385(7):577-579. doi:10.1056/NEJMp2109285 34192429

[26] Alwan NA. The road to addressing long COVID. Science. 2021;373(6554):491-493. doi:10.1126/science.abg7113 34326224

[27] Berger Z, Altiery DE Jesus V, Assoumou SA, Greenhalgh T. Long COVID and health inequities: the role of primary care. Milbank Q. 2021;99(2):519-541. doi:10.1111/1468-0009.12505 33783907 PMC8241274

[28] Malik P, Patel K, Pinto C, . Post-acute COVID-19 syndrome (PCS) and health-related quality of life (HRQoL)—a systematic review and meta-analysis. J Med Virol. 2022;94(1):253-262. doi:10.1002/jmv.27309 34463956 PMC8662132

[29] Price BM. Long COVID, cognitive impairment, and the stalled decline in disability rates. Accessed October 3, 2022. https://www.federalreserve.gov/econres/notes/feds-notes/long-covid-cognitive-impairment-and-the-stalled-decline-in-disability-rates-20220805.html

[30] American Association for Public Opinion Research. Standard definitions: final dispositions of case codes and outcome rates for surveys, 9th ed. Accessed August 16, 2022. https://www-archive.aapor.org/AAPOR_Main/media/publications/Standard-Definitions20169theditionfinal.pdf

[31] Long SK, Kenney GM, Zuckerman S, . The health reform monitoring survey: addressing data gaps to provide timely insights into the Affordable Care Act. Health Aff (Millwood). 2014;33(1):161-167. doi:10.1377/hlthaff.2013.0934 24352654

[32] Kyle MA, Frakt AB. Patient administrative burden in the US health care system. Health Serv Res. 2021;56(5):755-765. doi:10.1111/1475-6773.13861 34498259 PMC8522562

[33] Shartzer A, Long SK, Anderson N. Access to care and affordability have improved following Affordable Care Act implementation; problems remain. Health Aff (Millwood). 2016;35(1):161-168. doi:10.1377/hlthaff.2015.0755 26674536

[34] Ayoubkhani D, Pawelek P, Gaughan C. Technical article: updated estimates of the prevalence of post-acute symptoms among people with coronavirus (COVID-19) in the UK: 26 April 2020 to 1 August 2021. Accessed August 18, 2022. https://www.ons.gov.uk/peoplepopulationandcommunity/healthandsocialcare/conditionsanddiseases/articles/technicalarticleupdatedestimatesoftheprevalenceofpostacutesymptomsamongpeoplewithcoronaviruscovid19intheuk/26april2020to1august2021

[35] Centers for Disease Control and Prevention National Center for Health Statistics. 2022 National Health Interview Survey questionnaire. Accessed August 18, 2022. https://ftp.cdc.gov/pub/Health_Statistics/NCHS/Survey_Questionnaires/NHIS/2022/EnglishQuest-508.pdf

[36] US Office of Management and Budget. Standards for Maintaining, Collecting, and Presenting Federal Data on Race and Ethnicity. Federal Register. Published October 30, 1997. Accessed February 15, 2023. https://www.govinfo.gov/content/pkg/FR1997-10-30/pdf/97-28653.pdf

[37] US Census Bureau. Measuring racial and ethnic diversity for the 2020 census. Accessed March 9, 2023. https://www.census.gov/newsroom/blogs/random-samplings/2021/08/measuring-racial-ethnic-diversity-2020-census.html

[38] Urban Institute. Health reform monitoring survey—survey resources. Accessed August 17, 2022. https://www.urban.org/policy-centers/health-policy-center/projects/health-reform-monitoring-survey/survey-resources

[39] Goda GS, Soltas EJ. The impact of COVID-19 illnesses on workers. Accessed February 15, 2023. https://www.nber.org/papers/w30435

[40] Agarwal SD, Sommers BD. Insurance coverage after job loss—the importance of the ACA during the COVID-associated recession. N Engl J Med. 2020;383(17):1603-1606. doi:10.1056/NEJMp2023312 32813967

[41] Benjamini Y, Hochberg Y. Controlling the false discovery rate: a practical and powerful approach to multiple testing. J R Stat Soc Series B Stat Methodol. 1995;57(1):289-300. doi:10.1111/j.2517-6161.1995.tb02031.x

[42] Cha AE. She went to one doctor, then another and another. *The Washington Post*. Accessed October 3, 2022. https://www.washingtonpost.com/health/2022/04/18/long-covid-medical-care-challenge/

[43] Albarracín D, Bedford T, Bollyky T, . Getting to and sustaining the next normal: a roadmap for living with COVID. The Rockefeller Foundation. Accessed October 4, 2022. https://www.rockefellerfoundation.org/wp-content/uploads/2022/03/Getting-to-and-Sustaining-the-Next-Normal-A-Roadmap-for-Living-with-Covid-Report-Final.pdf

[44] Davenport K. COVID “long haulers” can carry additional burden of getting insurers to cover care. Georgetown University Center on Health Insurance Reforms. Accessed October 4, 2022. https://chirblog.org/covid-long-haulers-can-carry-additional-burden-getting-insurers-cover-care/

[45] Houston M. Prior authorization—boon or bane: federal and state policymakers seek reforms to insurers’ utilization management practices. Georgetown University Center on Health Insurance Reforms. Accessed October 3, 2022. https://chirblog.org/prior-authorization-boon-bane-federal-state-policymakers-seek-reforms-insurers-utilization-management-practices/

[46] Pollitz K, Lo J, Wallace R, Mengistu S. Claims denials and appeals in ACA marketplace plans in 2020. Kaiser Family Foundation. Accessed October 3, 2022. https://www.kff.org/private-insurance/issue-brief/claims-denials-and-appeals-in-aca-marketplace-plans/

[47] Koumpias AM, Schwartzman D, Fleming O. Long-haul COVID: healthcare utilization and medical expenditures 6 months post-diagnosis. BMC Health Serv Res. 2022;22(1):1010. doi:10.1186/s12913-022-08387-3 35941617 PMC9358916

[48] Tolbert J, Artiga S, Kates J, Rudowitz R. Implications of the lapse in federal COVID-19 funding on access to COVID-19 testing, treatment, and vaccines. Kaiser Family Foundation. Accessed October 3, 2022. https://www.kff.org/coronavirus-covid-19/issue-brief/implications-of-the-lapse-in-federal-covid-19-funding-on-access-to-covid-19-testing-treatment-and-vaccines/

[49] Centers for Medicare & Medicaid Services. Re: mandatory Medicaid and CHIP coverage of COVID-19-related treatment under the American Rescue Plan Act of 2021. Accessed October 5, 2022. https://www.medicaid.gov/federal-policy-guidance/downloads/sho102221.pdf

[50] Davis HE, McCorkell L, Vogel JM, Topol EJ. Long COVID: major findings, mechanisms and recommendations. Nat Rev Microbiol. 2023;21(3):133-146. doi:10.1038/s41579-022-00846-2 36639608 PMC9839201

[51] O’Hare AM, Vig EK, Iwashyna TJ, ; VA COVID Observational Research Collaboratory (CORC). Complexity and challenges of the clinical diagnosis and management of long COVID. JAMA Netw Open. 2022;5(11):e2240332. doi:10.1001/jamanetworkopen.2022.40332 36326761 PMC9634500

[52] McGrath LJ, Scott AM, Surinach A, Chambers R, Benigno M, Malhotra D. Use of the postacute sequelae of COVID-19 diagnosis code in routine clinical practice in the US. JAMA Netw Open. 2022;5(10):e2235089. doi:10.1001/jamanetworkopen.2022.35089 36201207 PMC9539719

[53] Thompson EJ, Williams DM, Walker AJ, ; OpenSAFELY Collaborative. Long COVID burden and risk factors in 10 UK longitudinal studies and electronic health records. Nat Commun. 2022;13(1):3528. doi:10.1038/s41467-022-30836-0 35764621 PMC9240035

