# Supplementary material for: Health Care Access and Affordability Among US Adults Aged 18 to 64 Years With Self-reported Post–COVID-19 Condition
Source: JAMA Netw Open. 2023 Apr 10;6(4):e237455. doi: 10.1001/jamanetworkopen.2023.7455 (PMC10087049; doi:10.1001/jamanetworkopen.2023.7455)
Supplement: Supplement 1. — eTable 1. Demographic and Geographic Characteristics of Health Reform Monitoring Survey Respondents and Nonrespondents eTable 2. Logistic Regression Models Estimating Association of Post–COVID-19 Condition With Unmet Needs for Health Care in Past 12 Months Because of Costs eTable 3. Estimated Probabilities of Health Care Access Outcomes eTable 4. Estimated Probabilities of Health Care Access Outcomes Using Bootstrapped Standard Errors eReferences. [file jamanetwopen-e237455-s001.pdf]

Supplemental Online Content

Karpman M, Zuckerman S, Morriss S. Health care access and affordability among US adults aged 18 to 64 years with self-reported post–COVID-19 condition. *JAMA Netw Open*. 2023;6(4):e237455. doi:10.1001/jamanetworkopen.2023.7455

eTable 1. Demographic and Geographic Characteristics of Health Reform Monitoring Survey Respondents and Nonrespondents

eTable 2. Logistic Regression Models Estimating Association of Post–COVID-19 Condition With Unmet Needs for Health Care in Past 12 Months Because of Costs

eTable 3. Estimated Probabilities of Health Care Access Outcomes

eTable 4. Estimated Probabilities of Health Care Access Outcomes Using Bootstrapped Standard Errors

eReferences.

This supplemental material has been provided by the authors to give readers additional information about their work.

© 2023 Karpman M et al. *JAMA Network Open.*

eTable 1. Demographic and Geographic Characteristics of Health Reform Monitoring Survey Respondents and Nonrespondents

Characteristic	Respondents (%)[a] (N=9484)	Nonrespondents (%)[a] (N=9678)[b]	P value[c]
Sex			
Female	49.8	56.9	<.001
Male	50.2	43.1	<.001
Age, y			
18-49	57.9	71.2	<.001
50-64	42.1	28.8	<.001
Race and ethnicity			
Asian	3.8	3.1	0.01
Black	12.0	15.5	<.001
Hispanic	18.7	27.4	<.001
White	60.6	49.2	<.001
Other[d]	5.0	4.8	0.60
Educational attainment			
High school degree or less	31.4	35.3	<.001
Some college	30.1	35.5	<.001
Bachelor's degree or more	38.5	29.1	<.001
Residence in a rural area[e]	14.1	14.2	0.78

[a] Sample sizes and percentage estimates are unweighted. Percentage estimates are rounded.

[b] Nonrespondents include 9563 individuals who did not take the survey, 105 individuals who were excluded because of high levels of item nonresponse, and 10 individuals who were excluded from analysis because they did not answer questions on diagnosed COVID-19 or post-COVID-19 condition.

© 2023 Karpman M et al. *JAMA Network Open.*

[c] P values were estimated using independent sample *t* tests of the differences between respondents and nonrespondents.

[d] Other race and ethnicity includes adults who did not identify as Hispanic and who identified as American Indian or Alaska Native, Native Hawaiian or other Pacific Islander, more than one race, or some other race.

[e] Adults who lived in a rural area include those who did not live in a metropolitan statistical area.

© 2023 Karpman M et al. *JAMA Network Open.*

eTable 2. Logistic Regression Models Estimating Association of Post–COVID-19 Condition With Unmet Needs for Health Care in Past 12 Months Because of Costs

	(1)			(2)			(3)		
	coefficient (log odds)	standard error		coefficient (log odds)	standard error		coefficient (log odds)	standard error	
Reported current Long COVID (ref.)									
Ever diagnosed with COVID-19 but without Long COVID	-0.545	0.084	*	-0.544	0.085	*	-0.564	0.085	*
Never diagnosed with COVID-19	-0.602	0.075	*	-0.608	0.079	*	-0.620	0.083	*
Female sex	0.340	0.054	*	0.282	0.053	*	0.275	0.057	*
Male (ref.)									
Age 18-34	0.361	0.094	*	0.304	0.094	*	0.293	0.094	*
Age 35-49	0.260	0.068	*	0.278	0.067	*	0.288	0.067	*
Age 50-64 (ref.)									
Asian	-0.705	0.305	*	-0.696	0.290	*	-0.582	0.250	*
Black	0.027	0.087		-0.070	0.083		-0.107	0.082	
Hispanic	0.191	0.097		0.073	0.103		0.122	0.112	
White (ref.)									
Other race or ethnicity[a]	0.233	0.142		0.139	0.138		0.169	0.147	
High school degree or less	0.261	0.088	*	-0.019	0.093		-0.024	0.089	
Some college	0.348	0.091	*	0.136	0.091		0.128	0.094	
Bachelor's degree or more (ref.)									
Citizen	0.051	0.146		0.096	0.139		0.065	0.159	
Not a citizen (ref.)									
Married	-0.207	0.056	*	-0.069	0.067		-0.062	0.063	
Not married (ref.)									
Lives with dependent children under 19	0.256	0.061	*	0.118	0.061		0.087	0.060	
Does not live with dependent children under 19 (ref.)									
Ever diagnosed with hypertension	0.209	0.092	*	0.184	0.089	*	0.167	0.089	
Ever diagnosed with hyperlipidemia	0.179	0.114		0.179	0.114		0.193	0.117	
Ever diagnosed with coronary heart disease, angina, heart attack, or other heart condition	0.432	0.160	*	0.401	0.158	*	0.388	0.155	*
Ever diagnosed with stroke	0.142	0.195		0.135	0.192		0.129	0.187	
Ever diagnosed with cancer	0.121	0.163		0.154	0.166		0.170	0.174	
Ever diagnosed with diabetes	0.245	0.080	*	0.207	0.077	*	0.210	0.075	*
Ever diagnosed with asthma	0.316	0.086	*	0.318	0.084	*	0.341	0.081	*
Ever diagnosed with COPD, emphysema, or chronic bronchitis	0.270	0.181		0.236	0.172		0.185	0.168	

© 2023 Karpman M et al. *JAMA Network Open.*

Ever diagnosed with arthritis, gout, lupus, or fibromyalgia	0.469	0.088	*	0.432	0.088	*	0.421	0.090	*
Diagnosed with multiple physical health conditions	0.083	0.153		0.075	0.150		0.053	0.147	
Not diagnosed with physical health conditions (ref.)									
Insured all months of the year	-0.958	0.093	*	-1.361	0.144	*	-1.294	0.153	*
Insured some months but not every month	0.135	0.117		-0.204	0.136		-0.146	0.142	
Uninsured all months of the year (ref.)									
Lives in urban area (ref.)									
Lives in rural area[b]	0.069	0.106		-0.006	0.114		-0.040	0.123	
Employer-sponsored health insurance at time of survey (ref.)									
Public health insurance at time of survey				-0.333	0.128	*	-0.318	0.128	*
Private nongroup or unspecified type of health insurance at time of survey				0.009	0.103		-0.009	0.096	
Uninsured at time of survey				-0.672	0.165	*	-0.675	0.164	*
Family income less than or equal to 138% of federal poverty level (FPL)				1.100	0.112	*	1.100	0.111	*
Family income greater than 138% to 249% FPL				0.993	0.104	*	0.992	0.103	*
Family income greater than 250% to 399% FPL				0.800	0.099	*	0.781	0.100	*
Family income greater than or equal to 400% FPL (ref.)									
Lives in Northeast region (ref.)									
Lives in Midwest region							0.138	0.075	
Lives in South region							0.209	0.095	*
Lives in West region							-0.176	0.108	
Lowest quartile of population-to-primary care physician ratio (ref.)[c]									
Second quartile of population-to-primary care physician ratio							0.145	0.121	
Third quartile of population-to-primary care physician ratio							0.082	0.084	
Highest quartile of population-to-primary care physician ratio							0.078	0.121	
Constant	-1.181	0.219	*	-1.095	0.250	*	-1.254	0.254	*
Observations	9,484			9,484			9,422		

* P<0.05

[a] Other race and ethnicity includes adults who do not identify as Hispanic and who identify as American Indian or Alaska Native, Native Hawaiian or Other Pacific Islander, more than one race, or some other race.

[b] Adults who live in a rural area include those who do not live in a metropolitan statistical area.

[c] Population-to-primary care physician ratio is based on data from the County Health Rankings[1] and originally derived from 2019 American Medical Association Physician Masterfile data included in the 2020-2021 Area Health Resources File. Estimates exclude 62 observations with missing data.

© 2023 Karpman M et al. *JAMA Network Open.*

eTable 3. Estimated Probabilities of Health Care Access Outcomes

	(1) Main model		(2) With added controls for family income and health insurance coverage type		(3) With added controls for Census region and population-to-primary care physician ratio	
	predicted probability	standard error	predicted probability	standard error	predicted probability	standard error
**Has a usual place to get health care**						
Adults with current Long COVID	0.828	0.016	0.828	0.016	0.825	0.017
Adults ever diagnosed with COVID-19 but without Long COVID	0.835	0.011	0.835	0.011	0.835	0.011
Adults never diagnosed with COVID-19	0.803	0.010	0.803	0.010	0.803	0.010
**Did not get needed care in past 12 months for the following reasons:**						
Costs						
Adults with current Long COVID	0.270	0.019	0.268	0.020	0.268	0.021
Adults ever diagnosed with COVID-19 but without Long COVID	0.183	0.012	0.184	0.012	0.182	0.013
Adults never diagnosed with COVID-19	0.175	0.010	0.175	0.010	0.174	0.010
Difficulty finding a provider accepting new patients						
Adults with current Long COVID	0.164	0.010	0.164	0.010	0.164	0.010
Adults ever diagnosed with COVID-19 but without Long COVID	0.101	0.007	0.103	0.007	0.103	0.007
Adults never diagnosed with COVID-19	0.107	0.006	0.106	0.005	0.106	0.005
Difficulty getting an appointment as soon as needed						
Adults with current Long COVID	0.220	0.014	0.220	0.014	0.222	0.014
Adults ever diagnosed with COVID-19 but without Long COVID	0.144	0.006	0.146	0.006	0.146	0.006
Adults never diagnosed with COVID-19	0.139	0.005	0.138	0.005	0.137	0.005
Difficulty getting to a doctor's office or clinic when it was open						
Adults with current Long COVID	0.110	0.011	0.110	0.011	0.110	0.011
Adults ever diagnosed with COVID-19 but without Long COVID	0.053	0.004	0.054	0.004	0.054	0.004
Adults never diagnosed with COVID-19	0.067	0.002	0.067	0.002	0.067	0.002

© 2023 Karpman M et al. *JAMA Network Open.*

Difficulty getting a telehealth visit						
Adults with current Long COVID	0.076	0.011	0.077	0.011	0.078	0.012
Adults ever diagnosed with COVID-19 but without Long COVID	0.031	0.003	0.032	0.003	0.032	0.003
Adults never diagnosed with COVID-19	0.040	0.003	0.040	0.003	0.040	0.003
Difficulty finding transportation						
Adults with current Long COVID	0.061	0.009	0.063	0.009	0.064	0.010
Adults ever diagnosed with COVID-19 but without Long COVID	0.035	0.004	0.038	0.004	0.038	0.004
Adults never diagnosed with COVID-19	0.051	0.004	0.049	0.004	0.049	0.004
Difficulty getting health plan authorization for care or prescription drugs						
Adults with current Long COVID	0.166	0.010	0.166	0.010	0.166	0.010
Adults ever diagnosed with COVID-19 but without Long COVID	0.108	0.006	0.110	0.006	0.109	0.006
Adults never diagnosed with COVID-19	0.103	0.005	0.102	0.005	0.102	0.005
Difficulty finding a doctor accepting coverage type						
Adults with current Long COVID	0.159	0.012	0.159	0.012	0.159	0.012
Adults ever diagnosed with COVID-19 but without Long COVID	0.096	0.006	0.100	0.006	0.099	0.006
Adults never diagnosed with COVID-19	0.100	0.005	0.098	0.005	0.098	0.005
Difficulty getting information on network, covered services, or cost of care						
Adults with current Long COVID	0.136	0.013	0.137	0.013	0.139	0.013
Adults ever diagnosed with COVID-19 but without Long COVID	0.076	0.005	0.077	0.005	0.075	0.005
Adults never diagnosed with COVID-19	0.086	0.006	0.085	0.006	0.085	0.007
**Additional health care affordability problems:**						
Problems paying family medical bills in past 12 months						
Adults with current Long COVID	0.235	0.021	0.233	0.021	0.234	0.021
Adults ever diagnosed with COVID-19 but without Long COVID	0.159	0.012	0.160	0.012	0.158	0.013
Adults never diagnosed with COVID-19	0.147	0.010	0.147	0.010	0.146	0.010
Currently has medical bills that are past due						
Adults with current Long COVID	0.238	0.025	0.236	0.025	0.236	0.025
Adults ever diagnosed with COVID-19 but without Long COVID	0.157	0.016	0.159	0.016	0.158	0.017
Adults never diagnosed with COVID-19	0.140	0.012	0.140	0.012	0.139	0.012

© 2023 Karpman M et al. *JAMA Network Open.*

eTable 4. Estimated Probabilities of Health Care Access Outcomes Using Bootstrapped Standard Errors

	(1) Main model		
	predicted probability	standard error	bootstrapped standard error
**Has a usual place to get health care**			
Adults with current Long COVID	0.828	0.016	0.017
Adults ever diagnosed with COVID-19 but without Long COVID	0.835	0.011	0.011
Adults never diagnosed with COVID-19	0.803	0.010	0.009
**Did not get needed care in past 12 months for the following reasons:**			
Costs			
Adults with current Long COVID	0.270	0.019	0.017
Adults ever diagnosed with COVID-19 but without Long COVID	0.183	0.012	0.012
Adults never diagnosed with COVID-19	0.175	0.010	0.010
Difficulty finding a provider accepting new patients			
Adults with current Long COVID	0.164	0.010	0.009
Adults ever diagnosed with COVID-19 but without Long COVID	0.101	0.007	0.007
Adults never diagnosed with COVID-19	0.107	0.006	0.005
Difficulty getting an appointment as soon as needed			
Adults with current Long COVID	0.220	0.014	0.014
Adults ever diagnosed with COVID-19 but without Long COVID	0.144	0.006	0.007
Adults never diagnosed with COVID-19	0.139	0.005	0.005
Difficulty getting to a doctor's office or clinic when it was open			
Adults with current Long COVID	0.110	0.011	0.011
Adults ever diagnosed with COVID-19 but without Long COVID	0.053	0.004	0.005
Adults never diagnosed with COVID-19	0.067	0.002	0.003
Difficulty getting a telehealth visit			
Adults with current Long COVID	0.076	0.011	0.010
Adults ever diagnosed with COVID-19 but without Long COVID	0.031	0.003	0.003

© 2023 Karpman M et al. *JAMA Network Open.*

Adults never diagnosed with COVID-19	0.040	0.003	0.003
Difficulty finding transportation			
Adults with current Long COVID	0.061	0.009	0.009
Adults ever diagnosed with COVID-19 but without Long COVID	0.035	0.004	0.004
Adults never diagnosed with COVID-19	0.051	0.004	0.003
Difficulty getting health plan authorization for care or prescription drugs			
Adults with current Long COVID	0.166	0.010	0.011
Adults ever diagnosed with COVID-19 but without Long COVID	0.108	0.006	0.006
Adults never diagnosed with COVID-19	0.103	0.005	0.004
Difficulty finding a doctor accepting coverage type			
Adults with current Long COVID	0.159	0.012	0.013
Adults ever diagnosed with COVID-19 but without Long COVID	0.096	0.006	0.006
Adults never diagnosed with COVID-19	0.100	0.005	0.005
Difficulty getting information on network, covered services, or cost of care			
Adults with current Long COVID	0.136	0.013	0.013
Adults ever diagnosed with COVID-19 but without Long COVID	0.076	0.005	0.005
Adults never diagnosed with COVID-19	0.086	0.006	0.006
**Additional health care affordability problems:**			
Problems paying family medical bills in past 12 months			
Adults with current Long COVID	0.235	0.021	0.021
Adults ever diagnosed with COVID-19 but without Long COVID	0.159	0.012	0.012
Adults never diagnosed with COVID-19	0.147	0.010	0.009
Currently has medical bills that are past due			
Adults with current Long COVID	0.238	0.025	0.022
Adults ever diagnosed with COVID-19 but without Long COVID	0.157	0.016	0.016
Adults never diagnosed with COVID-19	0.140	0.012	0.011

Bootstrapped standard errors are based on the standard deviation of estimated predicted probabilities across 100 replications using bootstrap resampling weights.

© 2023 Karpman M et al. *JAMA Network Open.*

eReferences

[1] County Health Rankings & Roadmaps 2022. University of Wisconsin Population Health Institute. Accessed February 22, 2023. https://www.countyhealthrankings.org.

© 2023 Karpman M et al. *JAMA Network Open.*
