# Supplementary material for: Health Care Access and Affordability Among US Adults Aged 18 to 64 Years With Self-reported Post–COVID-19 Condition
Source: JAMA Netw Open. 2023 Apr 10;6(4):e237455. doi: 10.1001/jamanetworkopen.2023.7455 (PMC10087049; doi:10.1001/jamanetworkopen.2023.7455)
Supplement: Supplement 2. — Data Sharing Statement [file jamanetwopen-e237455-s002.pdf]

Data Sharing Statement

Karpman. Health Care Access and Affordability Among US Adults Aged 18 to 64 Years With Self-reported Post–COVID-19 Condition. *JAMA Network Open*. Published online April 10, 2023.
doi:10.1001/jamanetworkopen.2023.7455

Data

Data available: Yes
Data types: Deidentified participant data
How to access data: The public use data file for the 2022 Health Reform Monitoring Survey will be made available through the Health and Medical Care Archive of the Inter-university Consortium for Political and Social Research (ICPSR): https://www.icpsr.umich.edu/web/pages/HMCA/index.html . Public use files are deposited with ICPSR nine months after the survey is fielded.
When available: beginning date: 04-05-2023

Supporting Documents

Document types: Other (please specify)
Additional Information: Codebook
How to access documents: The codebook will be made available through the Health and Medical Care Archive of the Inter-university Consortium for Political and Social Research (ICPSR): https://www.icpsr.umich.edu/web/pages/HMCA/index.html .
When available: beginning date: 04-05-2023

Additional Information

Who can access the data: The public use file will be made available to anyone requesting the data.
Types of analyses: The data will be available for any types of analyses.
Mechanisms of data availability: The data will be made available without investigator support.

© 2023 Karpman M et al. *JAMA Network Open*.
